# Neurobiological Mechanisms and Therapeutic Potential of Glucagon-like Peptide-1 Receptor Agonists in Binge Eating Disorder: A Narrative Review

**DOI:** 10.3390/ijms262210974

**Published:** 2025-11-13

**Authors:** Sujitra Tongta, Titiwat Sungkaworn, Nutthapoom Pathomthongtaweechai

**Affiliations:** 1 Faculty of Medicine Ramathibodi Hospital, Mahidol University, Bangkok 10400, Thailand; 2 Chakri Naruebodindra Medical Institute, Faculty of Medicine Ramathibodi Hospital, Mahidol University, Samut Prakan 10540, Thailand

**Keywords:** GLP-1, binge eating disorder, reward system, dopamine, eating disorders, semaglutide, liraglutide

## Abstract

Binge eating disorder (BED) is a prevalent eating disorder lacking adequate pharmacological interventions. This review examines the therapeutic potential of glucagon-like peptide-1 receptor agonists (GLP-1RAs), medications approved for type 2 diabetes and obesity now being investigated for eating disorders through their modulation of metabolic and reward pathways. A narrative review was conducted using PubMed/MEDLINE, through May 2025, to examine GLP-1RA effects on BED, including preclinical and clinical studies, mechanistic investigations, and relevant reviews. GLP-1 receptors (GLP-1Rs) are expressed in hypothalamic nuclei, regulating energy homeostasis and mesolimbic circuits controlling food reward. Preclinical studies demonstrate that GLP-1RAs reduce food-seeking behavior, suppress dopamine signaling in reward circuits, and modulate neural transmission in key brain regions. These effects extend beyond appetite suppression to directly modify reward processing underlying compulsive eating. Emerging clinical evidence with semaglutide and liraglutide report reductions in binge eating episodes, decreased food cravings, and improved symptom scores. However, current studies remain small-scale with methodological limitations, and translating findings from animal models to human eating disorder complexity presents significant challenges. This review integrates preclinical and clinical evidence demonstrating that GLP-1RAs modulate both metabolic and reward pathways. By elucidating the underlying neurobiological mechanisms, GLP-1RAs may offer advantages over current symptom-focused therapies for BED.

## 1. Introduction

Feeding and eating disorders (FEDs) are a growing health burden that significantly impairs both mental and physical health [1]. Among the deadliest of all mental illnesses, they are attributable to severe medical complications and suicide [2,3,4]. As defined by the Diagnostic and Statistical Manual of Mental Disorders (DSM-5) and the International Classification of Diseases (ICD-11), FEDs are characterized by persistent disturbances in eating-related behavior that compromise biopsychosocial functioning [5].

Multiple factors contribute to these disorders, including genetic, psychological, social, and biological influences [6]. The most prevalent FEDs are anorexia nervosa (AN), bulimia nervosa (BN), and binge-eating disorder (BED) [7,8].

BED is the most prevalent eating disorder, with lifetime prevalence estimates of approximately 2.8% in women and 1.0% in men, with systematic reviews reporting rates reaching up to 6% [3,9,10]. These estimates exceed those for anorexia nervosa (1.4% in women, 0.2% in men) and bulimia nervosa (1.9% in women, 0.6% in men) [3,9], establishing BED as the most common eating disorder among adults [11]. The global lifetime prevalence of FEDs has increased notably over the past two decades, particularly affecting women [3]. Moreover, BED is now recognized to occur across the lifespan rather than being limited to youth populations, with binge eating rates of 3.5–12% in women aged 50 and older, and up to 26% experiencing weekly episodes in some populations [12,13]. BED is characterized by recurrent episodes of consuming large quantities of food in a short period, accompanied by feelings of loss of control [14,15,16]. While BED is the formal clinical diagnosis, the core behavior of binge eating (BE) can occur across multiple eating disorder presentations. Beyond its high prevalence, BED presents urgent clinical needs. The disorder is associated with significant medical and psychiatric comorbidity, including high rates of anxiety, depression, and obsessive–compulsive disorder, as well as medical complications affecting multiple organ systems from cardiovascular damage and neurological impairment to bone density loss [4,17,18].

Current first-line treatment relies on psycho-behavioral therapy, such as cognitive behavioral therapy (CBT) and interpersonal therapy (IPT) [19,20,21]. However, these interventions are frequently associated with high relapse rates and limited long-term outcomes [22,23]. Furthermore, pharmacological interventions are insufficient, and specialized treatment for BED remains difficult to access with inconsistent efficacy and significant adverse effects [17,21]. These limitations show unmet clinical needs, leading to the investigation of innovative therapeutic strategies that target the underlying neurobiological mechanisms.

The pathophysiology of BED demonstrates notable parallels with substance use disorders, suggesting shared dysregulated brain reward circuits [15]. This mechanistic similarity, combined with BED’s higher prevalence compared to AN and BN, significant comorbidity burden, and limited approved pharmacological treatments, creates urgent clinical needs [3,9,17,18]. Furthermore, BED’s neurobiological profile of dysregulated reward-driven overconsumption aligns with GLP-1RA effects on both appetite regulation and reward circuits [24]. Unlike AN’s complex food avoidance behaviors and body image pathology, BED’s core pathophysiology of reward-driven overconsumption makes it a particularly suitable target for GLP-1RA intervention and novel therapies targeting reward pathways [25]. This has led to the investigation of innovative therapeutic strategies targeting underlying neurobiological mechanisms. The hormone glucagon-like peptide-1 (GLP-1), a key mediator in the gut–brain axis, has emerged as a promising therapeutic target due to its dual role in regulating both energy balance and reward pathways [26,27]. GLP-1 receptors (GLP-1Rs) are uniquely positioned in both hypothalamic nuclei regulating energy balance and reward-related dopaminergic areas, including the nucleus accumbens (NAc) and ventral tegmental area (VTA) [28,29,30].

GLP-1 receptor agonists (GLP-1RAs), including semaglutide and liraglutide, are already approved for treating type 2 diabetes and obesity [31,32,33,34,35,36,37,38,39]. They are known to modulate brain reward pathways and reduce food cravings, which are core features of eating disorders [40,41,42]. Growing evidence supports their potential therapeutic application in BED, with recent attention focused on their ability to reduce binge eating episodes [43].

This narrative review will explore the compelling rationale for investigating the GLP-1 system in BED, detailing its biological basis and analyzing the promising therapeutic evidence from preclinical and clinical studies. Through narrative synthesis of the existing literature, this work aims to provide a comprehensive overview of the current therapeutic implications and potential of GLP-1RAs to combat BED and identify key areas for future research.

A comprehensive literature search was conducted using PubMed/MEDLINE from inception through May 2025 to identify studies examining GLP-1RA effects on reward-related eating behavior and BED. The search strategy employed combinations of GLP-1-related terms (‘GLP-1’, ‘glucagon-like peptide-1’, ‘GLP-1 receptor agonist’, ‘semaglutide’, ‘liraglutide’, ‘exendin-4’, ‘dulaglutide’) with eating behavior and neurobiological terms (‘binge eating disorder’, ‘BED’, ‘eating disorder’, ‘feeding behavior’, ‘reward system’, ‘dopamine’, ‘mesolimbic’, ‘food intake’). We included preclinical studies examining GLP-1RA effects on feeding behavior and reward circuits, clinical trials and pilot studies evaluating GLP-1RAs in eating disorders, and mechanistic studies on GLP-1 receptor signaling in reward-related brain regions. The search was limited to English-language publications, with additional relevant articles identified through citation searching. Data were synthesized using a narrative approach, organizing findings thematically according to neurobiological mechanisms, preclinical evidence, and clinical studies. This integrative synthesis emphasizes mechanistic understanding of how GLP-1RAs modulate reward circuits and eating behavior in BED, providing a comprehensive framework for evaluating their therapeutic potential. This narrative review synthesizes the current evidence on GLP-1RA mechanisms and therapeutic potential in BED through comprehensive analysis of the neurobiological basis and clinical findings.

## 2. GLP-1 Therapeutic Opportunity in Eating Disorders

The pathophysiology of FEDs is multifactorial, involving complex interactions among genetic, psychological, and environmental factors that contribute to dysregulated eating and loss of control [44,45]. Recent research has revealed additional factors, such as gut microbiome dysbiosis, correlating with dysregulated eating behaviors [46]. A core feature of FEDs involves fundamental disruption of feeding control from imbalances between two primary, interconnected systems, namely the homeostatic pathway, which regulates energy balance, and the hedonic pathway, which controls motivated food consumption [47,48]. Eating disorders are characterized by dysfunction in the integration of homeostatic and hedonic systems, where hedonic drives dominate physiological hunger and satiety signals, contributing to loss of control over eating [1]. BED appears to be common among eating disorders in older populations, with a notable proportion being late-onset cases [49,50], suggesting that these dysfunctional patterns can emerge across the lifespan and may involve distinct etiological pathways.

The homeostatic system, centered in the hypothalamus, regulates energy balance by integrating peripheral signals through key nuclei, including the arcuate nucleus (ARC), paraventricular nucleus (PVN), and lateral hypothalamus (LH) [51,52,53,54]. The ARC contains both appetite-stimulating (orexigenic) neurons that co-express neuropeptide Y (NPY) and agouti-related peptide (AgRP), and appetite-suppressing (anorexigenic) proopiomelanocortin (POMC) neurons [55,56]. These hypothalamic circuits serve as the central hub for the gut–brain axis, integrating both short- and long-term signals [57,58,59]. Long-term energy status is communicated through leptin, secreted by adipose tissue in proportion to fat stores to suppress appetite, and insulin, secreted by pancreatic beta cells to regulate glucose metabolism and signal metabolic state [60]. Meanwhile, short-term satiety is regulated by gastrointestinal hormones released in response to food intake. These include the satiety-promoting L-cell hormones GLP-1 and peptide YY (PYY), cholecystokinin (CCK) released by I-cells to stimulate digestive enzymes and promote satiety, and ghrelin, produced by gastric cells to stimulate hunger and initiate food intake [61,62,63]. These peripheral signals reach the brain through both humoral pathways via circumventricular organs (CVO), which lack a complete blood–brain barrier and allow peripheral hormones direct access to the central nervous system circuit, and direct neural communication via the vagus nerve [64,65,66,67]. Specifically, gut-secreted GLP-1 modulates vagal neural pathways in with signals transmitted to the nucleus tractus solitarius (NTS) in the brainstem and relayed to hypothalamic nuclei, while centrally secreted GLP-1 from the NTS projects widely to create a comprehensive feedback loop that aligns food intake with energy demands [68,69]. In contrast, the hedonic system is a reward-based system driving the compulsive consumption of highly palatable foods, often independent of energy needs through the mesolimbic dopaminergic reward circuit [47,70,71]. This pathway comprises dopaminergic neurons projecting from the VTA of the midbrain to the NAc in the ventral striatum, where dopamine signals food reward value and drives motivation [72]. The reward circuitry interacts closely with the mesocortical dopamine pathway, which connects the VTA to key cortical regions, including the orbitofrontal cortex (OFC), ventromedial prefrontal cortex (vmPFC), medial prefrontal cortex (mPFC), and anterior cingulate cortex (ACC) [73,74]. These regions are essential for higher-order cognitive functions, such as inhibitory control, decision-making, and assessing long-term consequences, all of which are frequently impaired in eating disorders [74,75].

These homeostatic and hedonic systems are interconnected through direct hypothalamic-mesolimbic connections. During fasting states, decreased glucose levels activate glutamate LH neurons that project to and excite VTA dopaminergic neurons, driving food-seeking behavior [76,77]. Conversely, reward signals from highly palatable foods can activate mesolimbic circuits that override normal hypothalamic satiety cues, creating persistent motivation to continue eating despite physical fullness [78]. The NAc sends feedback signals to both the LH and VTA through inhibitory connections, creating bidirectional control that normally stops eating when sufficient reward is received [79,80,81]. This integrated network allows metabolic needs and reward motivation to influence each other [82]. However, in eating disorders, this balance becomes dysregulated, where hedonic drives consistently override physiological hunger and satiety signals [83,84]. Dysregulation of these dopaminergic, glutamatergic, and GABAergic systems plays a significant role in eating disorder pathophysiology. The reward circuit operates through wanting and liking components [85,86]. Dopamine signaling within the mesolimbic pathway mediates motivational drive and craving for palatable food, while endogenous opioids and endocannabinoids mediate the pleasurable experience of consuming food [87,88,89]. In eating disorders, dysregulation occurs where dopamine-driven wanting becomes uncoupled from the liking or satiety response, contributing to compulsive food pursuit without corresponding satisfaction [83].

GLP-1 is uniquely positioned to address feeding dysregulation through its dual role in both homeostatic and hedonic systems [90]. GLP-1 acts centrally by activating POMC neurons in the hypothalamic ARC to promote satiety while simultaneously inhibiting dopamine neurons in the VTA to reduce food reward value [91,92,93]. GLP-1Rs are widely expressed throughout reward-related brain areas, and their activation significantly influences dopamine levels by modulating GABAergic and glutamatergic neurons [82,94]. Importantly, dysregulation of endogenous GLP-1 signaling may contribute to eating disorder pathophysiology, with studies showing altered GLP-1 secretion in eating disorders [95,96,97]. This evidence provides a mechanistic basis for targeting the GLP-1 system to correct feeding dysregulation, offering a comprehensive approach that addresses both metabolic and reward-driven aspects of disordered eating behaviors.

## 3. The Neurobiological and Molecular Basis of GLP-1 Action

### 3.1. GLP-1 Receptor Biology

The GLP-1R first gained significant attention in the field of endocrinology and diabetes due to its primary function as an incretin hormone, which enhances glucose-dependent insulin secretion and regulates systemic glucose homeostasis [98]. GLP-1 exerts its diverse physiological effects by activating GLP-1R [99]. The GLP-1R belongs to the class B G-protein coupled receptor (GPCR) family, characterized by a seven alpha-helical transmembrane domain and a large N-terminal extracellular domain crucial for binding to its ligands, including both GLP-1 and its pharmacological agonists.

Upon activation, the intracellular C-terminal tail of the receptor couples to the stimulatory G-protein (G_s_) subunit, triggering a canonical signaling cascade that involves the activation of adenylyl cyclase (AC) and a subsequent increase in intracellular cyclic adenosine monophosphate (cAMP) [100,101,102,103]. The resulting cAMP increase activates protein kinase A (PKA), a key downstream mediator of GLP-1 signaling that phosphorylates various target proteins, leading to increased glucose-dependent insulin secretion and modulated neuronal excitability [104,105]. GLP-1R activation can also engage non-canonical signaling pathways, including the phospholipase C (PLC) pathway, which triggers protein kinase C (PKC) activation, diacylglycerol (DAG) production, and intracellular calcium mobilization [106,107]. These diverse signaling events allow the GLP-1R to exert its pleiotropic effects across different cell types and tissues [108]. The receptor signaling is also regulated by desensitization and internalization, which are mechanisms that prevent over-stimulation and allow for recycling, which are important for sustained therapeutic effects [109,110].

The wide-ranging physiological effects of GLP-1RAs are fundamentally determined by the widespread distribution of GLP-1Rs throughout both peripheral tissues and the central nervous system. In the periphery, GLP-1Rs are found in multiple key metabolic organs, including the pancreas, gut, heart, and kidneys, where they mediate glucose-dependent insulin secretion, gastric emptying, and cardiovascular and renal regulation [98,111,112,113,114,115]. Within the central nervous system, GLP-1Rs play a pivotal role in both homeostatic and hedonic feeding control [30]. In homeostatic centers, GLP-1Rs are expressed in hypothalamic nuclei, including ARC, PVN, and LH, where they promote satiety and reduce food intake [116]. GLP-1Rs are abundant in the brainstem, particularly in the NTS, which integrates visceral signals from the gut via the vagus nerve, and the area postrema (AP), a CVO that responds directly to circulating GLP-1 [69,117]. Extending to the mesolimbic reward system, GLP-1R expression in the VTA and NAc facilitates direct modulation of dopaminergic signaling and attenuation of food reward [118,119]. Additional receptor distribution in the amygdala, hippocampus, and prefrontal cortex (PFC) allows GLP-1 to influence emotional, memory, and executive control aspects of feeding behavior [120].

### 3.2. Mechanisms of GLP-1 Receptor Agonist Action on Feeding Behavior

GLP-1RAs exert their therapeutic effects through both peripheral and central mechanisms, acting as a physiological brake on both appetite and reward-driven food consumption. Understanding the distinction between these pathways is crucial for comprehending their therapeutic potential in eating disorders.

#### 3.2.1. Peripheral Mechanisms of Action

The endogenous GLP-1 system originates peripherally in the gastrointestinal tract, primarily from enteroendocrine L-cells located in the ileum and colon in response to nutrient ingestion [121]. These specialized cells release GLP-1 in response to nutrient ingestion, with a major stimulus coming from the fermentation of dietary fiber by the gut microbiome, which produces short-chain fatty acids (SCFAs), such as acetate, butyrate, and propionate [122,123]. These SCFAs activate specific GPCRs on the L-cells, triggering the release of GLP-1 and establishing a crucial link between gut microbiota metabolism and hormonal appetite regulation [124,125,126]. As an incretin hormone, GLP-1 plays a fundamental role in glucose homeostasis by stimulating glucose-dependent insulin secretion from pancreatic beta-cells and inhibiting glucagon release, which is the primary mechanism by which GLP-1RAs treat type 2 diabetes [127,128].

GLP-1RAs are approved by the U.S. Food and Drug Administration for managing type 2 diabetes and obesity through multiple mechanisms, including enhanced insulin secretion, glucagon suppression, delayed gastric emptying, and modulation of central appetite pathways [98]. In type 2 diabetes, GLP-1RAs reduce HbA1c by 0.8–1.5% depending on agent and dosage [129], with semaglutide demonstrating superior glycemic control and greater reductions in body weight compared to other GLP-1RAs [130]. Comparative analyses show GLP-1RAs provide superior metabolic efficacy versus other antidiabetic therapies, with sustained benefits for up to two years [131]. For weight management, liraglutide 3.0 mg induces approximately 5% body weight reduction, while semaglutide 2.4 mg achieves 12–15% weight loss over 52–68 weeks [132,133,134].

Beyond these metabolic effects, GLP-1RAs have multiple peripheral actions that contribute to feeding regulation, including slowing gastric emptying, which promotes prolonged satiety by contributing to feelings of fullness and decreasing the rate of intestinal nutrient absorption with subsequent reduction in post-meal glucose spikes [135,136]. Additionally, GLP-1RAs act on vagal afferent nerve endings in the gut wall, signaling satiety to the brainstem, and influence the myenteric plexus of the enteric nervous system [127,137,138].

#### 3.2.2. Central Mechanisms of Action

While FDA-approved GLP-1RAs, such as semaglutide and liraglutide, do not readily cross the blood–brain barrier, they can access central nervous system regions through CVO [139,140,141]. Studies using fluorescently labeled semaglutide and liraglutide have demonstrated drug accumulation in multiple brain areas, including regions typically protected by the blood–brain barrier, such as the NTS and AP [142,143,144].

In homeostatic centers, they activate anorexigenic POMC neurons in the ARC while inhibiting orexigenic NPY and AgRP neurons [145,146,147]. GLP-1 acts centrally by activating POMC neurons in the hypothalamic ARC to promote satiety while simultaneously inhibiting dopamine neurons in the VTA to reduce homeostatic hunger and decrease the food reward value [91,92,93]. Electrophysiological studies have confirmed direct neuronal responses and corresponding changes in gene expression [91,143,148].

Beyond homeostatic control, GLP-1RAs directly modulate reward processing through the mesolimbic system. The NAc serves as a central integration node, receiving dopaminergic input from the VTA and glutamatergic input from prefrontal cortical regions [149,150]. GLP-1Rs are widely expressed throughout these reward-related brain areas, and their activation significantly influences dopamine levels by modulating GABAergic and glutamatergic neurons [82,94]. This leads to reduced dopamine release in the NAc, thereby attenuating the motivational drive to consume palatable foods [151,152].

In brainstem regions, particularly the NTS and AP, GLP-1RAs reinforce satiety signals transmitted from the gut via the vagus nerve [69]. This comprehensive neuroanatomical distribution permits GLP-1RAs to target both homeostatic hunger signals and hedonic food motivation simultaneously. The integration of peripheral incretin effects, homeostatic appetite control, and central reward processing establishes GLP-1RAs as promising therapeutic tools for eating disorders. This integrated mechanism enables GLP-1RAs to bridge metabolic and neuropsychiatric treatment approaches, targeting both physiological and behavioral aspects of eating disorders.

## 4. Therapeutic Potential of GLP-1R Agonists for Binge-Eating Disorder

### 4.1. Preclinical Models and Baseline Findings for Binge Eating Research

Rodent models have been developed to provide insights into reward-related eating behaviors, which play a significant role in eating disorders. To study these complex human conditions, researchers have established animal models that mimic specific behavioral components of BED. According to the DSM-5, BE is defined as consuming an unusually large amount of food in a discrete period of time, such as a 2-h period, coupled with a sense of a lack of control during the episode [153]. BN combines BE episodes with inappropriate compensatory behaviors like self-induced vomiting [154]. As vomiting behavior cannot be replicated, rodent models focus primarily on mimicking binge-type eating and its underlying motivations [155].

Several methods are used to induce binge-eating behavior in rodents, including the manipulation of food availability, diet composition, feeding schedules, and stress exposure. Brief periods of food restriction can trigger binge-eating behavior [155]. Nevertheless, this model may not fully replicate human BED since the motivation is primarily hunger-driven. Another relevant approach provides intermittent access to highly palatable foods, such as sucrose, which drives increased intake patterns similar to binge eating after periods of deprivation [25,156]. Additionally, stress exposure can trigger binge-like episodes in rats, particularly when palatable food is available post-stress [157]. Both acute and chronic stressors, such as fasting or foot shock, can induce these behaviors when rats are given access to palatable food following stress exposure [157]. Among these approaches, stress-induced models most closely resemble human BED [158,159]. These paradigms employ restraint or foot-shock stress combined with palatable food access, producing robust, reproducible binge-like eating patterns [160]. They capture key BED features including stress-triggered episodes and emotional dysregulation, providing superior construct validity compared to other approaches. While intermittent access models induce intake escalation and progressive ratio schedules assess motivation, stress-induced paradigms uniquely integrate the affective disturbances and episodic nature of human BED, making them most relevant for therapeutic evaluation.

To comprehensively understand the mechanisms driving these behaviors, several validated methods assess binge and reward-related paradigms. Quantitative assessments include measuring caloric intake and comparing the amount of palatable food consumed to standard chow [161]. Behavioral measures, such as latency to feed, provide insight into compulsivity, as binge-eating rats often demonstrate increased impulsivity [162]. Since eating disorders involve dysregulated reward pathways, operant conditioning models are used to measure the drive to obtain food [163]. The progressive ratio (PR) schedule of reinforcement is widely used, where rodents have to make an increasing number of responses to earn a reward, such as pressing levers for sucrose. The breakpoint represents the highest ratio an animal completes and indicates how motivated the animal is to obtain the reward [164]. Conditioned place preference (CPP) represents a complementary approach using classical conditioning to assess learned preferences for a rewarding stimulus. In this paradigm, animals learn to associate a chamber with palatable food rewards, and the time subsequently spent in that chamber reflects the learned reward value of the food [165,166]. These models are complementary, as PR focuses on the motivational drive or wanting, while CPP addresses the hedonic component or liking of reward.

These validated preclinical models have revealed important neurobiological alterations that characterize binge-type eating behaviors. In animals exhibiting binge-eating patterns, repeated episodes of palatable food intake are associated with reduced adenosine A2A receptor expression in the amygdala and VTA, as well as downregulation of dopamine D2 receptors in the NAc [167]. Additionally, neural activity in the NAc core and shell becomes hyper-responsive during initial binge episodes but less responsive during chronic episodes [168]. These changes reflect disruption in the mesolimbic dopamine pathway, a crucial circuit regulating reward-related behaviors and motivation [169].

Importantly, these findings parallel functional brain studies in humans, which demonstrate reduced dopamine release and functional disconnection between the frontal cortex and striatum in individuals with binge-eating behaviors [163,170]. These similarities between animal and human studies support the use of these preclinical models for understanding eating disorder mechanisms and testing potential treatments.

### 4.2. Effects of GLP-1 Receptor Agonists on Eating Behavior and Reward

GLP-1 receptor agonists have demonstrated therapeutic potential beyond their original applications in type 2 diabetes and obesity treatment. These medications are now being investigated for eating disorders characterized by compulsive overeating and dysregulated food reward processing. GLP-1RAs, including exendin-4, semaglutide, liraglutide, and dulaglutide, have shown effects on both homeostatic appetite regulation and hedonic feeding through their action on brain reward circuitry.

#### 4.2.1. Exendin-4

Exendin-4 (Ex-4) is a synthetic analog originally derived from the saliva of the Gila monster [171]. Ex-4 has been extensively studied in preclinical research due to its high binding affinity, prolonged half-life, and brain penetrance, making it especially suitable for investigating central mechanisms of feeding regulation [171,172]. Central administration into brain regions involved in reward and appetite regulation leads to significant suppression of palatable food intake, suggesting its role in modulating hedonic feeding behavior [28,173,174,175]. When administered into the NAc core of rats, Ex-4 could suppress μ-opioid receptor-mediated feeding [176]. Notably, sex-dependent responses were observed, with male rats showing enhanced motivation for palatable food compared to females following intra-LH administration [174]. In contrast, no significant sex differences were observed with intracerebroventricular infusion, suggesting that site-specific neural responses may account for these behavioral differences [175]. Ex-4 also influences dopaminergic activity. Administration into the NTS led to a four-fold increase in tyrosine hydroxylase mRNA expression in rats [28]. In the VTA, Ex-4 modulated dopamine signaling that was transiently activated in response to sucrose cues, indicating that it may attenuate cue-driven dopamine release associated with palatable food rewards [175,177]. Collectively, these findings highlight the therapeutic potential of Ex-4 to regulate maladaptive eating behavior through modulation of reward-related neural circuits.

#### 4.2.2. Semaglutide

Multiple studies demonstrate that semaglutide, a long-acting GLP-1 receptor agonist, suppresses appetite, reduces food cravings, and contributes to significant weight loss across both clinical and preclinical models [143,178,179,180]. In rodent models, peripheral semaglutide administration reliably decreased total food intake and dampened motivation for palatable foods, supporting its potential in addressing binge-type eating patterns [143]. Semaglutide modulates activity within reward-related dopaminergic circuits in complex ways. While semaglutide at a higher dose enhanced dopaminergic signaling during reward collection, the animals obtained fewer rewards [180]. This suggests a possible dissociation between reward signaling and food-seeking behavior. Interestingly, this contrasts with earlier findings from other GLP-1RAs like Ex-4, which suppressed dopamine release in the NAc [28,175]. These findings reflect different reward phases. GLP-1RAs like Ex-4 suppress cue-triggered dopamine surges that drive food-seeking, thereby reducing cravings. However, semaglutide preserved dopamine responses during actual consumption while reducing total rewards obtained. This allows GLP-1RAs to reduce binge eating by blocking food cravings while maintaining the pleasurable aspects of eating. Peripherally administered semaglutide activated neurons in CVO, particularly the NTS and AP, regions regulating both homeostatic and hedonic feeding [143]. This was supported by increased c-Fos expression, a marker of neuronal activation, primarily localized in the hindbrain [180]. Despite increased neural activity in these regions, semaglutide did not stimulate compensatory intake, suggesting its anorexigenic effects involve modulation of central reward and satiety signals without triggering aversive responses [180]. Clinical evidence from a retrospective study showed that both semaglutide monotherapy and combination therapy with anti-obesity medications (AOM) resulted in significantly greater reductions in Binge-Eating Scale (BES) scores compared with AOM alone, with no significant difference between monotherapy and combination approaches [181,182]. At present, only one study has directly examined the effects of semaglutide on binge eating. Additional studies are needed to better define its therapeutic potential and clarify the mechanisms involved.

#### 4.2.3. Liraglutide

Liraglutide is well-documented to produce anorectic effects in both rodent models and humans, leading to reduced caloric intake and body weight in several studies [183,184,185]. Beyond reducing hedonic food value, liraglutide enhances cognitive control over food-related behaviors, potentially through hippocampal-dependent mechanisms involved in behavioral inhibition [186,187,188]. With its ability to access brain regions, liraglutide acts on GLP-1 receptors in mesolimbic and mesocortical reward pathways, including the VTA and NAc, suppressing operant food-seeking behaviors [171,189]. Clinical trials provide mixed but encouraging results. In a randomized trial involving obese, non-diabetic individuals with BED, liraglutide led to significant reductions in BES scores, with 81% shifting to the non-binge category [190]. A pilot double-blind trial showed early reduction in binge episodes for both liraglutide and placebo groups, but liraglutide produced significantly greater weight loss, though interpretation was limited by medication misallocation [191]. A study examining liraglutide combined with behavioral therapy decreased Eating Disorder Examination Questionnaire (EDE-Q) scores and reduced binge episode frequency, although statistical significance declined over extended follow-up periods. The lack of a placebo control limited interpretation [40]. Among these three studies, two showed that higher doses of liraglutide (3 mg/day) were associated with greater reductions in weight, body mass index (BMI), and waist circumference, suggesting dose-dependent effects. Although current evidence is preliminary, liraglutide shows promise for BED management. Larger, well-controlled trials are needed to establish its efficacy and clarify its mechanisms.

#### 4.2.4. Dulaglutide

Dulaglutide has also attracted attention for its potential role in improving BED. Dulaglutide showed promise in a pilot study of patients with type 2 diabetes and BED, demonstrating significant reductions in BES scores accompanied by decreases in body weight, BMI, and body fat mass compared to gliclazide treatment [192].

Collectively, these findings highlight the therapeutic potential of GLP-1RAs across both preclinical and clinical settings. Table 1 and Table 2 summarize the preclinical and clinical evidence, respectively, demonstrating how different GLP-1RAs modulate reward-related feeding behaviors across various animal models and human study populations, administration routes, brain regions, and key findings.

### 4.3. Region-Specific GLP-1 Receptor Activation and Behavioral Outcomes

**Figure 1:**
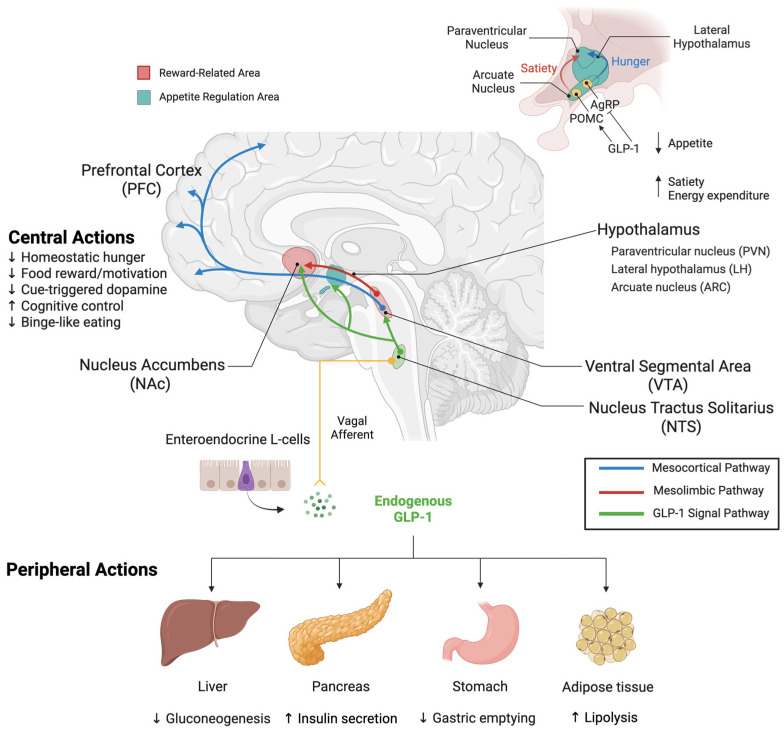
Integrated Peripheral and Central GLP-1 Modulation of Appetite and Reward Pathways. Peripheral Actions: GLP-1, an incretin hormone secreted by enteroendocrine L-cells in the intestinal tract, acts on multiple peripheral organs. In the pancreas, GLP-1RAs stimulate insulin secretion and inhibit glucagon release. In the stomach, they delay gastric emptying to promote satiety. In the liver, they regulate glucose metabolism. In adipose tissue, they modulate energy expenditure and metabolism. GLP-1 signals via vagal afferent nerves to the nucleus tractus solitarius (NTS) in the brainstem, establishing direct gut–brain communication. Central Actions and Reward Circuits: The NTS serves as a critical integration hub, both receiving peripheral GLP-1 signals and producing GLP-1 centrally. NTS GLP-1 neurons send projections to reward centers, including the VTA and NAc, and appetite-regulating nuclei in the hypothalamus. The mesolimbic reward circuit originates from dopaminergic neurons in the VTA that project to the NAc, regions critical for reward-related behaviors. Within the hypothalamus, key appetite-regulating regions include the LH, PVN, and ARC. In the ARC, GLP-1 activates POMC neurons to promote satiety while inhibiting AgRP neurons to suppress hunger. These integrated signals from reward and homeostatic centers project to the PFC, where feeding behavior and reward processing are coordinated. Through these coordinated peripheral and central mechanisms, GLP-1RAs modulate both metabolic function and reward-driven eating behavior. Color coding: Blue = mesocortical pathway (reward centers to PFC), red = mesolimbic dopaminergic pathway (VTA to NAc), green = central GLP-1 neuronal projections (NTS to hypothalamus and reward regions), yellow = peripheral GLP-1 signaling (intestinal L-cells to brainstem via vagal afferents). Arrows indicate neural projections and signaling pathways; (↑) indicates stimulatory effects, (↓) indicates inhibitory effects. Created with www.BioRender.com (accessed on 15 September 2025). Abbreviations: AgRP, agouti-related peptide; ARC, arcuate nucleus; GLP-1, glucagon-like peptide-1; LH, lateral hypothalamic area; NAc, nucleus accumbens; NTS, nucleus tractus solitarius; PFC, prefrontal cortex; POMC, pro-opiomelanocortin; PVN, paraventricular nucleus; VTA, ventral tegmental area.

The therapeutic effects of GLP-1RAs on eating behavior are rooted in their region-specific activation within the central nervous system. GLP-1Rs are widely expressed in multiple brain regions that are pivotal in modulating motivation, reward anticipation, and feeding inhibition. Key sites include the NTS, CVOs, several hypothalamic nuclei, and the mesolimbic reward pathway, particularly the VTA and NAc [28,30,194]. The hypothalamus integrates homeostatic hunger signals and interacts with reward circuits through key neurotransmitters, such as dopamine and serotonin [195], while the mesolimbic reward pathway mediates the hedonic aspects of feeding by responding to highly palatable food stimuli [194]. Figure 1 illustrates the anatomical and functional connectivity between GLP-1 signaling pathways and dopaminergic reward circuits involved in appetite regulation.

Direct administration of Ex-4 into the NTS suppressed palatable food intake in rats without affecting standard chow consumption and reduced operant responses for sucrose rewards, demonstrating selective suppression of hedonic feeding [28,196]. Central GLP-1R stimulation in the NTS also led to upregulation of tyrosine hydroxylase mRNA, a critical enzyme for dopamine synthesis, suggesting that GLP-1 influences dopaminergic tone within downstream reward circuits [28]. The NTS maintains important projections to key mesolimbic structures, including the VTA and NAc, creating an anatomical link through by which brainstem signaling can directly influence reward processing [197,198]. In the LH, which integrates both homeostatic and hedonic signals, acute Ex-4 administration reduced food-motivated behaviors, evident by decreased sucrose rewards earned and reduced lever presses [174]. This effect was sex-dependent, with a more pronounced response observed in male rats compared to females [199]. However, the broader literature reveals that female rodents generally show greater sensitivity to GLP-1RA effects on reward-driven eating [200]. This sex difference may reflect hormonal modulation of GLP-1R expression and function in reward circuits. Sex hormones influence GLP-1R levels [201], and estrogen enhances GLP-1RA effects on food reward behavior [202]. These findings suggest therapeutic responses may vary between sexes, with potential implications for sex-specific dosing or treatment strategies in BED.

**Figure 2:**
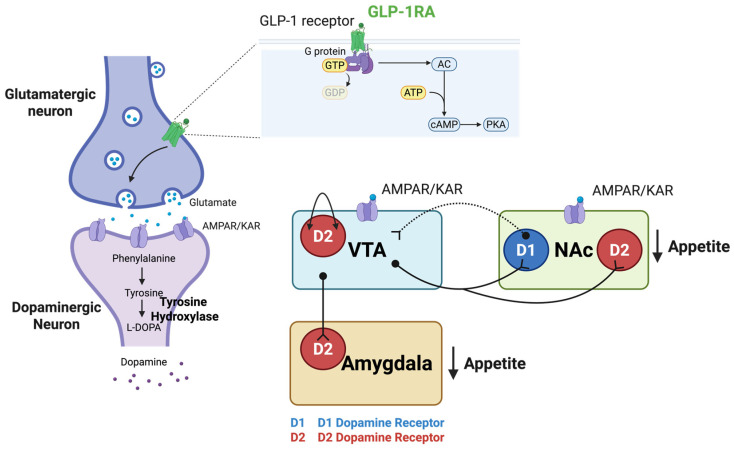
GLP-1 Receptor Signaling Mechanisms in Reward Circuits. GLP-1R activation modulates feeding behavior in key reward-related brain regions, including the VTA, NAc, and amygdala through GPCR signaling. GLP-1R stimulation increases cAMP and activates PKA, triggering intracellular signaling cascades that result in altered neurotransmitter release and synaptic transmission for appetite regulation. In the VTA and NAc, GLP-1R activation enhances presynaptic glutamate release, which stimulates postsynaptic AMPAR/KAR on target neurons. In the NAc, this glutamatergic mechanism activates D2-MSNs, contributing to appetite suppression through increased downstream inhibition of reward targets. Dopaminergic projections from the VTA to the NAc modulate reward-related behaviors, while GABAergic feedback from the NAc D1-MSNs to the VTA regulates dopaminergic neuron activity and modulates dopamine release to balance reward and motivational signals associated with food intake. D2 autoreceptors on VTA dopamine neurons provide additional negative feedback, further modulating dopamine release. VTA dopaminergic projections also innervate the amygdala, regulating emotional aspects of feeding behavior. Created with www.BioRender.com (accessed on 15 September 2025). Abbreviations: AMPAR/KAR, AMPA/kainate receptors; cAMP, cyclic adenosine monophosphate; D1-MSNs, dopamine D1 receptor-expressing MSNs; D2-MSNs, dopamine D2 receptor-expressing MSNs; GABA, gamma-aminobutyric acid; GLP-1R, glucagon-like peptide-1 receptor; GPCR, G protein-coupled receptor; MSNs, medium spiny neurons; NAc, nucleus accumbens; PKA, protein kinase A; VTA, ventral tegmental area.

GLP-1-dopamine crosstalk creates an integrated network modulating both homeostatic and hedonic feeding behaviors through complex, region-specific mechanisms. The mesolimbic pathway serves as the primary target for GLP-1-mediated reward modulation and is extensively innervated by GLP-1 neurons from the NTS [203]. This interaction extends to peripheral dopamine signaling in adipose tissue, where dopamine functions as a nutrient sensor and correlates with GLP-1R expression, suggesting coordinated metabolic regulation between central reward circuits and peripheral energy metabolism [136,204]. At the molecular level, GLP-1R activation initiates GPCR signaling that increases cAMP production and activates PKA, triggering intracellular cascades that alter neurotransmitter release and synaptic plasticity (Figure 2).

In the VTA, GLP-1R activation exhibits complex effects that may differ from expected dopaminergic outcomes. Ex-4 administration enhances presynaptic glutamatergic release via AMPA/kainate receptors (AMPA/KAR), increasing tyrosine hydroxylase expression and dopaminergic neuron firing [177]. However, this increased VTA activity preferentially directs dopamine release toward the amygdala rather than the NAc, where amygdala dopamine D2 receptor activation suppresses food intake [205]. This mechanism explains how increased VTA firing can ultimately suppress feeding behavior despite dopamine’s traditional role in reward enhancement. Within the VTA, D2 autoreceptors on dopamine neurons provide additional negative feedback that regulates dopamine release [206].

The NAc, consisting of core and shell subregions with distinct functional roles, responds differently to GLP-1R activation. The core governs goal-directed decision-making, including operant behaviors for palatable food, while the shell modulates incentive motivation driven by reward-predictive cues [89]. GLP-1R activation in the NAc enhances medium spiny neuron (MSN) activity through presynaptic glutamatergic signaling without directly affecting local dopamine levels [175,177]. This glutamatergic mechanism preferentially activates MSNs expressing dopamine D2 receptors (D2-MSNs) in the indirect pathway, increasing downstream inhibition of reward targets without directly affecting local dopamine levels, ultimately reducing consumption of rewarding substances [40,190,191]. The VTA sends dopaminergic projections to the NAc that regulate reward-related behaviors, while receiving inhibitory feedback from the NAc via GABAergic D1-MSNs, creating a bidirectional circuit that modulates dopaminergic activity [207,208].

Several preclinical studies consistently show that GLP-1RAs suppress dopamine responses to substance exposure. Peripheral Ex-4 reduces extracellular dopamine levels in the NAc after alcohol, nicotine, and cocaine intake, without affecting baseline dopamine levels, indicating that GLP-1R activation selectively inhibits substance-induced dopamine release while preserving normal dopaminergic tone [151,209]. Direct microinjection studies reveal regional specificity in GLP-1’s effects. Ex-4 administration into the VTA produces more potent suppression of motivated behavior than NAc administration, although both regions effectively reduce food intake when activated [173,203,210,211]. VTA injections significantly reduce motivated responding for palatable food and decrease NAc dopamine release, whereas NAc core activation reduces food intake and increases c-fos expression, indicating enhanced neural activity [203,212]. The mechanism extends beyond dopaminergic modulation to include glutamatergic signaling, as GLP-1R activation alters glutamate signaling in the NAc to promote negative energy balance without affecting dopamine activity [177].

Recent clinical studies support the therapeutic potential of GLP-1RAs in treating reward-related disorders. Semaglutide has been shown to suppress alcohol-induced dopamine elevation in the NAc, extending preclinical findings to clinically relevant medications [213]. Advanced formulations like the brain-penetrant agonist NLY01 demonstrate neuroprotective effects by directly targeting microglial GLP-1Rs, preventing dopaminergic neuron loss, and modulating astrocyte calcium signaling [214]. The consistency of dopamine suppression across different substances and GLP-1RA formulations demonstrates robust therapeutic mechanisms that position GLP-1RAs as promising therapeutic agents for disorders characterized by dysregulated reward processing, including BED, where the same mesolimbic circuits drive compulsive consumption of highly palatable foods.

### 4.4. Limitations of Current Research and Clinical Translation

Although GLP-1RAs have shown promise in preclinical rodent models of BED, several key limitations must be considered for clinical translation. BED is a complex psychiatric disorder involving multiple domains, including emotional regulation, cognitive control, and reward sensitivity, which are difficult to replicate in animal models. Rodent behavioral tests like CPP and operant conditioning offer quantitative insights. However, they cannot fully capture the complex psychological aspects of human BED. Biological and methodological variability further complicates interpretation. Different mouse strains exhibit inherent differences in baseline behaviors, with some strains showing higher anxiety-like behavior that may confound assessments of reward responsiveness [215]. Sex differences play a significant role, as studies using Ex-4 demonstrate that female rats show less potent intake suppression and reward-driven behavior compared to males, with significant effects observed only at higher doses [174]. The hormonal cycle can also modulate behavioral responses and drug efficacy, with effects more pronounced during the estrous phase [216]. Despite BED being more prevalent in females, insights into sex-specific drug responses remain underexplored [216]. Additionally, commonly used models employ lean or diet-induced obesity rodents, which may not fully replicate the neurobehavioral profile of human BED. Additionally, current preclinical research lacks aged animal models. While BED remains prevalent in older populations [12,13], studies evaluating GLP-1RA effects in aged rodents are scarce. Age-related changes in GLP-1R expression and dopaminergic function may influence therapeutic responses. Given BED’s clinical relevance in older adults, future preclinical research should incorporate aged animal models to optimize treatment strategies across the lifespan.

Preliminary clinical observations provide encouraging but limited evidence. A qualitative study of nine individuals prescribed GLP-1RAs reported reduced appetite, decreased consumption quantities, and improved mindfulness around food choices with corresponding decreases in EDE-Q scores. Two participants with compulsive eating behaviors, including one with a prior BED diagnosis, reported marked reductions in eating urges and improved control [193], with the BED participant achieving apparent remission. However, significant limitations exist in the current clinical research. Most studies are pilot trials with a small sample size and limited statistical power [217]. Study design challenges include inadequate blinding, drug misallocation, and study durations ranging from 11 to 52 weeks, which are potentially insufficient to capture long-term efficacy. Inconsistent dosing protocols, particularly for liraglutide, complicate cross-study comparisons. Assessment methodology remains problematic. Studies rely primarily on self-reported questionnaires, such as the EDE-Q and BES, which are subject to recall bias and underreporting [218]. Future research should integrate objective measures, including hormone levels, neuroimaging, or structured clinical assessments. Population heterogeneity, including comorbidities such as type 2 diabetes and psychiatric disorders, further complicates interpretation.

Clinical trial outcomes show considerable variability, with some studies reporting significant reductions in binge eating while others show non-significant trends. This heterogeneity likely reflects multiple factors. Small sample sizes (typically under 50 participants) limit statistical power [217]. Dosing protocols vary considerably, particularly for liraglutide, with higher doses generally showing stronger effects. Treatment duration ranges from 11 to 52 weeks, which may be insufficient for neurobiological adaptations. High placebo response rates occur in eating disorder trials. Population heterogeneity, including comorbidities such as type 2 diabetes and psychiatric disorders, further complicates interpretation [217,218]. These methodological limitations make it difficult to draw definitive conclusions about efficacy. Future research requires adequately powered, randomized controlled trials with standardized dosing protocols, objective assessment measures, and sufficient duration to establish true therapeutic benefits of GLP-1RAs in BED.

Emerging research explores GLP-1RA integration with established treatments. CBT remains the first-line non-pharmacological approach for BED, while lisdexamfetamine (LDX) is the only approved pharmacological agent [219]. A pilot study combining liraglutide with intensive behavioral therapy demonstrated reductions in binge eating episodes and improved metabolic outcomes [40,190]. Similarly, combining semaglutide with AOM improved BES scores, though this study found no significant difference between semaglutide monotherapy and dual therapy, suggesting that semaglutide alone may exert substantial therapeutic effects [181,182]. These findings highlight the potential for multidimensional approaches where GLP-1RAs complement existing therapies, though further trials are needed to explore the efficacy of the combination strategy.

GLP-1RAs and behavioral interventions may work synergistically through complementary mechanisms. GLP-1RAs reduce reward circuit hyperreactivity and hunger, creating favorable conditions for implementing CBT strategies. Prefrontal GLP-1R activation enhances cognitive control, facilitating cognitive restructuring, while hippocampal engagement accelerates extinction of maladaptive associations. Reduced cravings improve capacity to implement behavioral strategies, enhancing self-efficacy [40,190]. Sustained reward modulation may prevent post-CBT relapse [219]. This multi-level synergy provides rationale for combination therapy trials [181,182].

These limitations collectively call for well-designed, larger-scale, standardized studies integrating objective measures to evaluate both efficacy and safety of GLP-1RAs in BED treatment. Future research should prioritize proper study design with adequate sample sizes, standardized dosing protocols, longer follow-up periods, and objective assessment tools while accounting for population heterogeneity and sex-specific responses. Addressing these methodological challenges will be essential for translating promising preclinical and preliminary clinical findings into effective therapeutic interventions for binge eating disorder.

## 5. Conclusions and Future Perspectives

The GLP-1 system represents a promising therapeutic target for eating disorders through its dual modulation of metabolic and reward-driven behaviors. Preclinical evidence demonstrates that GLP-1RAs act on both homeostatic circuits in the hypothalamus and brainstem, as well as hedonic pathways in the mesolimbic reward system. By targeting key brain regions, including the NTS, VTA, and NAc, these agents effectively suppress operant behaviors for palatable food and attenuate food-motivated drive. This mechanism-based approach addresses core neurobiological drivers rather than isolated symptoms, providing a strong foundation for clinical applications in BED and BN. Despite promising preclinical findings, significant challenges remain. The translational gap between animal models and human eating disorders is substantial, as complex psychological symptoms cannot be fully captured in rodent paradigms. Critical research needs include larger placebo-controlled trials across different eating disorder subtypes, investigation of sex-specific effects given the high prevalence in women, and clarification of optimal dosing and treatment duration.

Future research should prioritize elucidating disorder-specific mechanisms across AN, BN, and BED while exploring combination therapies that integrate GLP-1RAs with behavioral interventions and other pharmacological agents. Additionally, advancing toward personalized medicine using neuroimaging and biomarker analysis could identify patients most likely to benefit from treatment. GLP-1RAs represent a promising new therapeutic avenue in eating disorder treatment by targeting fundamental neurobiological drivers of both homeostatic and hedonic feeding. This neurobiologically targeted approach offers the prospect of complementing existing treatments by addressing core pathophysiological mechanisms, potentially improving therapeutic outcomes in these complex disorders.

## Figures and Tables

**Table 1:** Preclinical Evidence for GLP-1RAs in Reward-Related Feeding Behaviors. Experimental parameters and behavioral/neurochemical outcomes from animal studies examining GLP-1RAs effects and underlying mechanisms.

Drug/Dosage/ Administration Route	Model	Key Findings	Reference
Exendin-4 - 0.05 and 0.10 µg - NAc core injection	Rats	- Decreased intake of sweetened fat diet when co-administered with μ-opioid receptor stimulation - No effect on sweetened fat diet intake when Ex-4 administered alone - Attenuated μ-opioid-induced hyperphagia—both Ex-4 doses reduced increased intake caused by μ-opioid receptor stimulation Possible Mechanism: Interaction between GLP-1 and opioid reward pathways—selective modulation of opioid-driven palatable food consumption	[176]
Exendin-4 - 0.05 and 0.15 μg - Lateral hypothalamus injection	Male and female rats	- Decreased food-motivated behavior—reduced sucrose rewards earned and fewer lever presses for sucrose without changes in inactive lever pressing - Decreased food intake - Suppressed reinforcement, intake and body weight in male rats - Less potent effects in female rats—sucrose reward suppression only at higher dose - Sex-divergent LH GLP-1R signaling—crucial for motivated behavior control in males but dispensable in females Possible Mechanism: Sex-specific LH GLP-1R pathways—controlling food motivation and intake regulation	[174]
Exendin-4 - 0.05 and 0.10 µg - Lateral ventricle injection	Rats	- Increased latency to first lick with higher dose Ex-4 via lateral ventricle in both male and female rats - Dopamine transients evoked by sucrose-predictive cue—specific cue-related dopamine activity observed - Suppressed average dopamine activity with Ex-4 treatment Possible Mechanism: Reduced cue-evoked dopamine signaling—affecting reward anticipation and feeding initiation	[175]
Exendin-4 - 0.05 and 0.10 μg - Intra NTS microinjection Lateral ventricle injection	Rats	- Reduced food intake—selective suppression of palatable food over chow - Decreased chow intake when offered as sole food source - Diminished operant responding for sucrose rewards Possible Mechanism: Enhanced dopamine synthesis—4-fold increase in tyrosine hydroxylase mRNA in VTA	[28]
Semaglutide - 1, 3, 10, 30, and 100 nmol/kg - Subcutaneous and intravenous injection	Diet-induced obese mice and Rats fed a high-fat diet	- Decreased body weight, fat mass, and food intake with minor effect on lean mass - Reduced chocolate intake preference—significant and dose-dependent reduction in body weight and energy intake driven by decreased palatable food consumption - Activated multiple brain regions—signaled circumventricular organs including AP, ME, OVLT, and SFO, as well as BBB-protected regions including LSc, SF, ARH, NTS, and DMX - Stimulated neuronal activity in appetite regulation areas with POMC/CART neuron depolarization and NPY/AgRP neuron hyperpolarization and inactivation Possible Mechanism: Glutamatergic pathway—differential activation of anorexigenic versus orexigenic neurons in hypothalamic appetite control circuits	[143]
Semaglutide - 0.1, 0.3, and 1 mg/kg - Intraperitoneal injection	Pitx3-cre mice with cre-dependent GCaMP6s virus into the VTA	- Decreased chow intake - Decreased number of sucrose rewards and reduced licks in the task - Increased VTA dopamine neuron activity during sucrose collection, independent of task performance - No alteration in VTA dopamine neuron activity during cue presentation - Reduced appetite and reward-seeking behavior while increasing VTA dopamine activity during reward consumption Possible Mechanism: Altered dopamine signaling—enhanced dopamine activity during reward consumption but not cue presentation may reduce overall reward-seeking behavior	[180]
Liraglutide 10 μg/kg - Intraperitoneal injection	Male and female rats	- No significant effects on body weight regardless of sex and diet intake - Enhanced inhibitory control—improved ability of inhibitory stimulus to suppress responding to reward cue with less responding when inhibitory light cue was present - Minimal effect on reward responding when inhibitory cue was absent Possible Mechanism: Hippocampal-dependent inhibitory processes that suppress feeding behavior	[186]

Abbreviations: Brain regions: AP, area postrema; ARH, arcuate nucleus of hypothalamus; BBB, blood–brain barrier; DMX, dorsal motor nucleus of the vagus; LSc, lateral septal nucleus, caudal part; ME, median eminence; NAc, nucleus accumbens; NTS, nucleus tractus solitarius; OVLT, organum vasculosum of the lamina terminalis; SF, septofimbrial nucleus; SFO, subfornical organ; VTA, ventral tegmental area. Peptides/Protein: AgRP, agouti-related peptide; CART, cocaine- and amphetamine-regulated transcript; NPY, neuropeptide Y; POMC, proopiomelanocortin.

**Table 2:** Clinical Evidence for GLP-1RAs in Reward-Related Feeding Behaviors. Experimental parameters and behavioral/neurochemical outcomes from animal studies examining GLP-1RAs effects and underlying mechanisms.

Drug/Dosage/ Administration Route	Subject/ Study Design	Key Findings	Reference
Semaglutide	BED Open-label retrospective cohort study (N = 98)	- Significantly greater BES score reduction in semaglutide group compared to AOM-only group - No significant difference in BES scores between semaglutide-only and semaglutide plus AOM groups - Semaglutide monotherapy appears effective—combination with AOM did not provide additional benefit Possible Mechanism: GLP-1 receptor-mediated appetite control—semaglutide effects independent of additional anti-obesity medication	[181]
Liraglutide - 1.8 mg/day - Subcutaneous injection	Non-diabetic obesity with subclinical binge eating Randomized controlled trial (N = 44; 12 weeks)	- Significant reductions in BES scores - Decreased body weight, BMI and waist circumference - Remarkable shift from binge to non-binge category Possible Mechanism: Improved appetite regulation and metabolic control—affecting central satiety pathways and peripheral metabolic parameters	[190]
Liraglutide - 3.0 mg/day - Subcutaneous injection	BMI ≥ 27 kg/m^2^ and BED Double-blind, randomized controlled trial (N = 27; 17 weeks)	- Reduced binge episode frequency with liraglutide treatment but no significant difference compared to placebo - Sharp decline in binge episodes in both groups after first week, remaining constant throughout 17-week trial - Greater weight loss - Larger reductions in BMI and waist circumference with liraglutide but without statistical significance Possible Mechanism: Appetite modulation—effects observed in both treatment and placebo groups suggest potential behavioral components	[191]
Liraglutide - 3.0 mg/day - Subcutaneous injection	Obesity Randomized controlled trial with exploratory analysis (N = 150; 52 weeks)	- Decreased food cravings - Reduced eating disorder psychopathology - Larger within-group decline in binge episodes at week 24 for IBT combined with liraglutide and multicomponent groups compared to IBT alone - Significant decrease in binge episodes - No significant between-group differences Possible Mechanism: Enhanced behavioral therapy effects—liraglutide may augment intensive behavioral therapy outcomes	[40]
Dulaglutide - 150 mg/week	Type 2 diabetes with BED Pilot open label, prospective controlled study (12 weeks)	- Significant decrease in body weight after 12 weeks of treatment - Reduced BMI and body fat mass - Improved BES scores indicating reduced binge eating severity Possible Mechanism: GLP-1 receptor activation—affecting both metabolic parameters and eating behavior regulation	[192]
Multiple GLP-1RAs Various Doses: Liraglutide - 0.6 to 3 mg once daily - Subcutaneous injection Semaglutide - 3 to 14 mg once daily - Oral administration Semaglutide - 0.25 mg to 1 mg once weekly - Subcutaneous injection Liraglutide - 0.6 mg to 1.8 mg once daily - Subcutaneous injection	Obesity and/or type 2 diabetes Qualitative, individual, semi-structured interviews (N = 9; 12–16 weeks)	- Reduced food consumption - Minimal change in food preference - Improved stress eating patterns - Decreased EDE-Q scores - Dramatic reduction in binge episodes - BED remission achieved Possible Mechanism: Improved eating behavior control—enhanced regulation of eating patterns and reduced stress-related food consumption	[193]

Abbreviations: AOM, anti-obesity medication; BED, binge eating disorder; BES, binge-eating scale; BMI, body mass index; EDE-Q, eating disorder examination questionnaire scores; IBT, intensive behavioral therapy.

## Data Availability

No new data were created or analyzed in this study. Data sharing is not applicable.

## Abbreviations

The following abbreviations are used in this manuscript:

AC	Adenylyl Cyclase
ACC	Anterior Cingulate Cortex
AgRP	Agouti-Related Peptide
AMPA/KAR	AMPA/Kainate Receptors
AN	Anorexia Nervosa
AOM	Anti-Obesity Medications
AP	Area Postrema
ARC	Arcuate Nucleus
BE	Binge Eating
BED	Binge Eating Disorder
BES	Binge-Eating Scale
BMI	Body Mass Index
BN	Bulimia Nervosa
cAMP	Cyclic Adenosine Monophosphate
CBT	Cognitive Behavioral Therapy
CCK	Cholecystokinin
CPP	Conditioned Place Preference
CVO	Circumventricular Organs
D1-MSN	Dopamine D1 Receptor-expressing Medium Spiny Neuron
D2-MSN	Dopamine D2 Receptor-expressing Medium Spiny Neuron
DAG	Diacylglycerol
DSM-5	Diagnostic and Statistical Manual of Mental Disorders, 5th Edition
EDE-Q	Eating Disorder Examination Questionnaire
Ex-4	Exendin-4
FEDs	Feeding and Eating Disorders
GLP-1	Glucagon-Like Peptide-1
GLP-1R	GLP-1 Receptor
GLP-1RA	GLP-1 Receptor Agonist
GPCR	G-Protein Coupled Receptor
ICD-11	International Classification of Diseases, 11th Revision
IPT	Interpersonal Therapy
LDX	Lisdexamfetamine
LH	Lateral Hypothalamus
mPFC	Medial Prefrontal Cortex
MSN	Medium Spiny Neuron
NAc	Nucleus Accumbens
NPY	Neuropeptide Y
NTS	Nucleus Tractus Solitarius
OFC	Orbitofrontal Cortex
PFC	Prefrontal Cortex
PKA	Protein Kinase A
PKC	Protein Kinase C
PLC	Phospholipase C
POMC	Proopiomelanocortin
PR	Progressive Ratio
PVN	Paraventricular Nucleus
PYY	Peptide YY
SCFA	Short-Chain Fatty Acid
vmPFC	Ventromedial Prefrontal Cortex
VTA	Ventral Tegmental Area
