# Correction: Tongta et al. Neurobiological Mechanisms and Therapeutic Potential of Glucagon-like Peptide-1 Receptor Agonists in Binge Eating Disorder: A Narrative Review. *Int. J. Mol. Sci.* 2025, *26*, 10974

**DOI:** 10.3390/ijms27052365

**Published:** 2026-03-03

**Authors:** Sujitra Tongta, Titiwat Sungkaworn, Nutthapoom Pathomthongtaweechai

**Affiliations:** 1 Faculty of Medicine Ramathibodi Hospital, Mahidol University, Bangkok 10400, Thailand; sujitra.ton@student.mahidol.edu; 2 Chakri Naruebodindra Medical Institute, Faculty of Medicine Ramathibodi Hospital, Mahidol University, Samut Prakan 10540, Thailand; titiwat.sun@mahidol.ac.th

In the original publication [1], there are 1 reference is retracted, which need to be corrected.

**Correction for updated references**

Reference 59 in the original manuscript has been retracted by the publisher and has been removed from the article.

**Text Correction** (Section 2, Paragraph 2):

*Original text:* “These hypothalamic circuits serve as the central hub for the gut–brain axis, integrating both short- and long-term signals [57–59].”

*Corrected text:* “These hypothalamic circuits serve as the central hub for the gut–brain axis, integrating both short- and long-term signals [57,58].”

**Reference List Correction:**

*Original Reference 59:* Ashique, S.; Mohanto, S.; Ahmed, M.G.; Mishra, N.; Garg, A.; Chellappan, D.K.; Omara, T.; Iqbal, S.; Kahwa, I. Gut-brain axis: A cutting-edge approach to target neurological disorders and potential synbiotic application. *Heliyon* **2024**, *10*, e34092. https://doi.org/10.1016/j.heliyon.2024.e34092.

*Correction:* Reference 59 has been removed from the reference list. All subsequent references have been renumbered (original references 60–219 are now references 59–218).

**Impact of Correction:**

The removal of this retracted reference does not affect the scientific conclusions of the article. The statements previously supported by this reference remain valid based on other cited literature and the primary findings of our meta-analysis. The authors apologize for inadvertently citing retracted literature and regret any confusion this may have caused. This correction was approved by the Academic Editor. The original publication has also been updated.
